# Comprehensive DNA Methylation Analysis Reveals a Common Ten-Gene Methylation Signature in Colorectal Adenomas and Carcinomas

**DOI:** 10.1371/journal.pone.0133836

**Published:** 2015-08-20

**Authors:** Árpád V. Patai, Gábor Valcz, Péter Hollósi, Alexandra Kalmár, Bálint Péterfia, Árpád Patai, Barnabás Wichmann, Sándor Spisák, Barbara Kinga Barták, Katalin Leiszter, Kinga Tóth, Ferenc Sipos, Ilona Kovalszky, Zoltán Péter, Pál Miheller, Zsolt Tulassay, Béla Molnár

**Affiliations:** 1 2nd Department of Medicine, Semmelweis University, Budapest, Hungary; 2 Molecular Medicine Research Group, Hungarian Academy of Sciences, Budapest, Hungary; 3 1st Department of Pathology and Experimental Cancer Research, Semmelweis University, Budapest, Hungary; 4 Tumor Progression Research Group, Hungarian Academy of Sciences, Budapest, Hungary; 5 Department of Gastroenterology and Medicine, Markusovszky University Teaching Hospital, Szombathely, Hungary; 6 Dana-Farber Cancer Institute, Harvard Medical School, Boston, Massachusetts, United States of America; Queen's University Belfast, UNITED KINGDOM

## Abstract

Microarray analysis of promoter hypermethylation provides insight into the role and extent of DNA methylation in the development of colorectal cancer (CRC) and may be co-monitored with the appearance of driver mutations. Colonic biopsy samples were obtained endoscopically from 10 normal, 23 adenoma (17 low-grade (LGD) and 6 high-grade dysplasia (HGD)), and 8 ulcerative colitis (UC) patients (4 active and 4 inactive). CRC samples were obtained from 24 patients (17 primary, 7 metastatic (MCRC)), 7 of them with synchronous LGD. Field effects were analyzed in tissues 1 cm (n = 5) and 10 cm (n = 5) from the margin of CRC. Tissue materials were studied for DNA methylation status using a 96 gene panel and for *KRAS* and *BRAF* mutations. Expression levels were assayed using whole genomic mRNA arrays. SFRP1 was further examined by immunohistochemistry. HT29 cells were treated with 5-aza-2’ deoxycytidine to analyze the reversal possibility of DNA methylation. More than 85% of tumor samples showed hypermethylation in 10 genes (*SFRP1*, *SST*, *BNC1*, *MAL*, *SLIT2*, *SFRP2*, *SLIT3*, *ALDH1A3*, *TMEFF2*, *WIF1*), whereas the frequency of examined mutations were below 25%. These genes distinguished precancerous and cancerous lesions from inflamed and healthy tissue. The mRNA alterations that might be caused by systematic methylation could be partly reversed by demethylation treatment. Systematic changes in methylation patterns were observed early in CRC carcinogenesis, occuring in precursor lesions and CRC. Thus we conclude that DNA hypermethylation is an early and systematic event in colorectal carcinogenesis, and it could be potentially reversed by systematic demethylation therapy, but it would need more in vitro and in vivo experiments to support this theory.

## Background

Colorectal cancer (CRC) is a clinically and molecularly heterogenous disease [1]. With over 1.2 million new cases annually, it is one of the most frequently occurring malignant diseases worldwide [2]. Despite considerable advances in the management of CRC, over 600,000 CRC-related deaths occur annually [2], reflecting a clear need for a better understanding of the disease.

The majority of CRCs arise via the adenoma-carcinoma sequence (ACS), which is thought to be driven by sequential accumulation of genetic mutations [3]. Recent whole genome sequencing studies confirmed the early findings that mutations in the *APC*, *KRAS*, and *P53* genes are frequent events in CRC development (81%, 41%, and 59%, respectively) [4]. Merely focusing on DNA mutations, however, do not fully depict the molecular complexity of the disease and consideration of epigenetic changes in CRC pathogenesis has become an intense area of focus.

Based on accumulating data, epigenetic changes and particularly alterations in the promoter DNA methylation are thought to be essential events in the pathogenesis of CRC [5]. It is known that chronic inflammation induces aberrant hypermethylation in a multitude of genes in different organs including the colon [6,7]. Surely, thousands of gene promoters, including many tumor suppressor genes may be hypermethylated in CRC, exceeding the number of genetic alterations [8]. The origin of such changes in DNA methylation remains an area of research. However, studies suggest 4 different molecular pathways that could lead to CRC [4,9].

A particular CRC subtype that is termed CpG island methlyator phenotype (CIMP) was first described in 1999 [10]. These CRCs arise from morphologically and molecularly distinct precursors, called serrated lesions and are predominantly located in the proximal colon [11]. They are characterized by *BRAF* V600E mutation and the hypermethylation of several genes [7]. In contrast with CRCs arising via the ACS, a subset of these cancers exhibit microsatellite instability (MSI) due to hypermethylation and subsequent underexpression of mismatch repair (MMR) genes (e.g. *MLH1*, *MSH2*, *MSH6*, *PMS2*), and this type of CRC confers a good prognosis. The frequency of sporadic MSI CRCs varies between geographical regions, but it represents a minority (10–15%) of CRCs, as most CRCs are microsatellite stable (MSS).

The analysis of DNA methylation was initially focused on the global levels of methylated cytosine. Digestion by methylation-sensitive restriction enzyme, bisulfite conversion and subsequent methylation sequencing and methylation-specific PCR all enable analysis of single, targeted genes [12]. Array technologies are suitable for parallel analysis of multiple gene promoters [13]. Most recently, bisulfite sequencing of the whole genome has been introduced, yet deeper insight to CRC-related methylation changes is still limited by the bioinformatic tools for remapping bisulfite converted, partially methylated sequences [12]. On the other hand, methods that are based on restricive enzymatic digestion can successfully overcome these obstacles [13].

In this study, our primary aim was to assess the changes in the DNA methylation profile and also to examine *KRAS* and *BRAF* mutations in the progression of normal epithelium to colorectal cancer. To determine whether DNA methylation field effect was present, we analyzed the normal gut epithelium, CRC precursor lesions and distinct CRCs, as well as the tissue adjacent to CRCs. In addition, we characterized the status of DNA methylation in our gene panel for ulcerative colitis (UC) patients, a model to ascertain the impact of chronic inflammation in the development of CRC. mRNA and protein expression levels were also characterized to validate the potential tumor suppressor role of selected hypermethylated genes.

## Materials and Methods

### Patient groups and ethics statements

Fresh fozen biopsy samples were collected by routine endoscopy and classified based on the histology; tubular adenoma with low-grade dysplasia (LGD, n = 17), adenoma with high-grade dysplasia (HGD, n = 6), CRC (n = 17), metastatic CRC (MCRC, n = 7), active UC (UCa, n = 4), inactive and long-standing UC (UCi, n = 4). As control, patients with normal histology were used. The normal biopsy samples were collected from adults (older than 18 years, N, n = 5) and young patients (younger than 18 years, Y, n = 5). For those patients who had normal histology, the indication for colonoscopy was screening for CRC in adults, and abdominal pain in children. Field effect was studied by taking samples from at least 10 cm (cancer normal, CN) and 1 cm away (field, F) from the macroscopically visible margin of the tumor. CRC patients were staged by the TNM system defined by the Union for International Cancer Control (UICC). Samples were taken distally from the splenic flexure, collected into RNAlater (Ambion, Life Technologies, Carlsbad, California, USA), and stored at −80°C until use. Biopsies from the same site were immediately fixed in buffered formalin for the histological evaluation.

This study was conducted according to the Helsinki declaration and it was approved by the local ethics committee and government authorities (Regional and Institutional Committee of Science and Research Ethics (TUKEB) Nr.: 69/2008 and 202/2009, Semmelweis University, Budapest, Hungary). Written informed consent was obtained in advance from all adult participants and from the next of kin, caretakers, or guardians on the behalf of the minors/children approved by the ethics committees.

### DNA isolation and methylation-sensitive restriction enzyme methylation array analysis

For DNA extraction, biopsy samples were homogenized in 2% sodium dodecyl sulphate, and digested with proteinase K (4 mg/mL) for 16 hours at 56°C. DNA was extracted using High Pure PCR Template Preparation Kit (Roche, Penzberg, Germany) as instructed by the manufacturer. DNA was eluted in 2x100 μl RNase- and DNase-free water and stored at -20°C. DNA methylation profiles were examined using the EpiTect Methyl qPCR Array System (Qiagen, Hilden, Germany). This method is based on the detection of the remaining DNA input after digestion by restriction enzyme, coupled with quantitative PCR. Treatment with a methylation-sensitive and a methylation-dependent restriction enzyme selectively digests unmethylated or methylated DNA, respectively, and treatment with both enzymes serves as a control for total DNA in the assay [14]. The relative fractions (%) of hypermethylated, intermediately methylated and unmethylated DNA are subsequently determined by comparing the amount of methylation in each digest with a mock digest. 96 genes were evaluated (S1 Table). The threshold for hypermethylation was set at 15%. Our aim was to present DNA methylation as a qualitative data (hypermethylated yes or no). With a threshold of 15% for hypermethylation, we could significantly differentiate between normal and tumorous tissue (S1 Fig). Reactions were performed according to the manufacturer’s protocol. Briefly, isolated DNA was incubated with either DNA methylation-dependent or sensitive methylases for 16 h at 37°C, then incubated at 65°C to halt methylase activity. Each 120 μL reaction contained 500 ng of genomic DNA. Following enzymatic digestion, samples were analyzed by fluorescence-based qPCR using LightCycler 480 (Roche). PCR conditions were as follows: 95°C for 10 min, 40 cycles of 97°C for 15 sec, 72°C for 1 min with realtime data acquisition. To ensure amplification of the desired products, high resolution melting (HRM) analysis was performed following the PCR reaction. The melting curve range was 65°–95°C, holding for 1 s at increments of 0.04°C for product detection.

After thermocycling was completed, C_T_ values were exported and 2^-ΔΔCT^ analysis was performed. Due to the inversely proportional relationship between threshold cycle and the amount of input DNA, and due to the doubling of PCR product with every cycle in the exponential phase of the reaction, the initial DNA amount in each digest before PCR was expressed as:

$$C_{Mo}=2^{-Ct(M_{o})};\;C_{Ms}=2^{-Ct(M_{s})};\;C_{Md}=2^{-Ct(M_{d})};\;C_{Msd}=2^{-Ct(M_{sd})}$$

The fraction of DNA in each digest was calculated by normalizing the DNA amount to the amount of digestible DNA. The amount of digestible DNA is equal to the total amount of DNA (determined from the mock digest) minus the amount of DNA resistant to DNA digestion (determined from the double digest).

Hypermethylated (HM) DNA Fraction:

$$F_{HM}=\frac{C_{Ms}}{C_{Mo}-C_{Msd}}=\frac{2^{-Ct(M_{s})}}{2^{-Ct(M_{o})}-2^{-Ct(M_{sd})}}$$

Unmethylated (UM) DNA Fraction:

$$F_{UM}=\frac{C_{Md}}{C_{Mo}-C_{Msd}}=\frac{2^{-Ct(M_{d})}}{2^{-Ct(M_{o})}-2^{-Ct(M_{sd})}}$$

The equations were formatted in MathType 6.9 (Design Science, Long Beach, CA, USA).

### Bisulfite PCR analysis and validation studies

Genomic DNA samples were bisulfite converted using EZ DNA Methylation-Direct Kit (Zymo Research, Irvine, CA, USA). To ensure efficient conversion, the maximum amount of input DNA did not exceed 500 ng per reaction, as suggested by the manufacturer. Eluted bisulfite modified DNA was quantified with Qubit 2.0 Fluorometer (Invitrogen, Life Technologies, Grand Island, NY, USA).

PCR and sequencing primers specific to relevant CpG island regions of previously selected genes indicated in CRC progression were designed using PyroMark Assay Design 2.0 (Qiagen) and BiSearch (Institute of Enzymology, Hungarian Academy of Sciences, Budapest, Hungary) software. CpG islands were identified using the UCSC Genome Browser database and CpG Plot (EMBOSS) software. PCR amplification and HRM were carried out sequentially on a LightCycler 480 instrument. Reaction volume was 15 μl and contained 1x AmpliTaq Gold PCR Master Mix (Applied Biosystems, Life Technologies), 0.25 μl ResoLight Dye (Roche), 1.5 mM MgCl_2_, 0.13 μM of each primer (Invitrogen), and 3 ng of bisulfite converted template. The amplification consisted of a denaturing step for 10 min at 95°C, followed by 9 cycles of touchdown from 65°C to 55°C with a ramp rate of 2.2°C, then 40 cycles of 95°C for 30 s, 55°C for 30 s, and 30 s 72°C. All

reactions were performed in duplicate. Primer sequences were: MAL-f GGAAAAATTGGGTTTTTAATTGGGGTTAG, MAL-r TTCAACTCCCTCTCATCTCCAAATCTC, (target area: chr2:95026139–95026340 on the hg38 genome, corresponding to 456–657 bp downstream from the transcriptional start), SFRP1-f ATTTAGGAGGTTGTAGGGTTGGA, SFRP1-r TTCCCCTTCTTTTTCTCCCCTTATC (target area: chr8:41309468–41309682 on the hg38 genome, corresponding to 2 to -213 bp from the transcriptional start, SFRP2-f GTTGTTAGAGAGGGGGATGTAAAGG, SFRP2-r ATACCACCCCAACACCAAAAAATTCCTAT (target area: chr4:153788387–153788549 on the hg38 genome, corresponding to 524–686 bp downstream from the transcriptional start. HRM analysis used a melting profile from 50 to 95°C with a ramp rate of 0.03°C/s and 20 acquisitions per °C. Unmethylated (0%) and methylated (100%) DNA standards were used from EpiTect Control DNA Kit (Qiagen).

### RNA extraction and whole genomic mRNA expression microarray analysis

Total RNA was extracted by using RNeasy Mini Kit according to the manufacturer’s instructions (Qiagen, Hilden, Germany). Whole genomic mRNA expression microarray analysis was performed previously by our group [15]. Briefly, biotinylated cRNA probes were synthesized fragmented using the One-Cycle Target Labeling and Control Kit (http://www.affymetrix.com/support/downloads/manuals/expression_s2_manual.pdf), according to the Affymetrix description. Two-cycle T7-based linear amplification was performed according to instructions of the manufacturer (Affymetrix Inc., Santa Clara, CA, USA). A volume of 10 mg of each fragmented cRNA sample was hybridized into HGU133 Plus2.0 array (Affymetrix) at 45°C for 16 h. Slides were washed and stained using Fluidics Station 450 and an antibody amplification staining method according to the manufacturer’s instructions. Fluorescent signals were detected by a GeneChip Scanner 3000 (Affymetrix). Data sets are available at the Gene Expression Omnibus database (GEO, http://www.ncbi.nlm.nih.gov/geo/) under access numbers: GSE4183, GSE10714, GSE15960 and GSE37364 [15–18].

### In vitro and in silico data

5-aza-2’-deoxycytidine treatment was performed as described previously [15,18.] HT29 colon adenocarcinoma cells (ATCC Number: HTB-38) were cultured at 37°C with 5% CO_2_ in RPMI-1640 medium (Sigma-Aldrich, St Louis, MO, USA) containing 10% FCS. In 25 cm^2^ cell culture flask, 1 500 000 cells/flask were cultured for 24 hours, then for demethylation the cells were treated with 10 μM 5-aza-2’-deoxycitidine (Sigma-Aldrich) for 72 hours in FCS-free medium. The treatment was carried out in 3 parallel experiments. In the control cultures PCR-grade water and acetate, the solvent of 5-aza-2’-deoxycitidine was added in 1:1 ratio. Whole genomic mRNA expression microarray analysis was performed as described above [15]. Data set is available at the Gene Expression Omnibus database (GEO, http://www.ncbi.nlm.nih.gov/geo/) under access number: GSE29060 [18]. Concerning molecular phenotype, HT29 cell line is MSS, KRAS wild type, BRAF V600E mutant [19]. For additional in silico analysis, microarray dataset using MSI-high, KRAS mutant, BRAF wild type CRC cell line HCT116, and re-expression array analysis by 5-aza-2’-deoxycytidine/Trichostatin A performed by an other research group was downloaded from the Gene Expression Omnibus (GEO) database (GSE14526) [20].

### Immunohistochemistry analysis of MMR proteins and SFRP1

Tissue microarrays (TMAs) were constructed with 2 mm cores from formalin-fixed and paraffin-embedded tissues. TMA sections were fixed on Superfrost Plus slides (VWRI, Leicestershire, UK) and incubated overnight at 37°C. Immunohistochemistry analysis was performed as previously described [21]. Following dewaxing and rehydration, endogenous peroxidase activity was blocked with 2.5% hydrogen peroxide in methanol for 30 minutes. The antigen retrieval was carried out in an electric pressure cooker for 40 minutes in TRIS-EDTA buffer (pH 9). After blocking in 1% bovine serum albumin, slides were incubated overnight at 58°C with monoclonal antibodies for MLH1 (1:80), MSH2 (1:80), MSH6 (1:80), PMS2 (1:150, all from Santa Cruz, California, USA), and SFRP1 (1:150, Abcam, Cambridge, UK). The antibody detection was carried out using the HISTOLS-MR-T kit (Hisztopatológia Kft., Pécs, Hungary) and visualized with 3,3'-diaminobenzidine Liquid and Substrate Chromogen System (Dako Cymation, Glostrup, Denmark). Slides were counterstained with haematoxylin, dehydrated with xylol, and mounted. After digital archiving (Pannoramic Flash 250 instrument, 3DHISTECH Ltd., Budapest, Hungary), stainings were evaluated with a Pannoramic Viewer digital microscope (software version 1.15; 3DHISTECH). A tumor was scored MSS if all four MMR proteins showed expression. SFRP1 protein expression was scored as follows: 0 (no immunoreaction), 1 (weak), 2 (moderate), and 3 (strong, diffuse cytoplasmic staining).

### *BRAF* and *KRAS* mutational analysis

All samples were analyzed for *BRAF* V600E and *KRAS* codon 12 and 13 mutations. *BRAF* mutation analysis was performed as previously described [22]. Briefly, 20 ng of genomic DNA and 12.5 μL of HotStarTaq Master Mix (Qiagen) was put in a final reaction volume of 25 μL. Thermal cycling conditions were 95°C for 12 min, followed by 40 cycles of 95°C for 10 s, 55°C for 20 s, and 72°C for 20 s. Primers used were this analysis were:

BRAF_fw_BIO: TCCAGACAACTGTTCAAACTGAT,

BRAF_rev: TGAAGACCTCACAGTAAAAATAGG,

BRAF_seq: TGATTTTGGTCTAGCTACA.

Targeted analysis of *BRAF* was performed by using pyrosequencing technology on a PyroMark Q24 instrument (Qiagen) and PyroMark Q24 software (Qiagen), following the manufacturer’s protocols. Activating *KRAS* mutations in codons 12 and 13 were tested by mutagenic PCR-RFLP analysis described elsewhere [23] with several modifications. Briefly, formalin-fixed, paraffin-embedded tissue sections were deparaffinized in xylene and ethanol. After drying, proteinase K digestion was performed overnight at 50°C, followed by heat inactivation at 94°C for 10 min. 10–20 ng unpurified DNA was amplified per PCR reaction. In addition, the final volume of 20 μl PCR mixture contained 1x ImmoMix Red (Bioline, London, UK) adjusted to 3.5 mM final MgCl_2_ concentration and 0.5 μM of each primer. Amplification was carried out in a Veriti 96-Well Fast Thermal Cycler (Applied Biosystems, Life Technologies) and the protocol was 10 min at 95°C, 10 cycles of 95°C for 40 s, 58°C for 40 s, and 72°C for 40 s, then 30 cycles of 86.5°C for 40 s, 58°C for 40 s, and 72°C for 40 s, and finally 72°C for 10 min. The primers used were as follows:

Mc12-f ACTGAATATAAACTTGTGGTAGTTGGACCT,

Mc12-r CTGTATCAAAGAATGGTCCTGGACCAGTA,

Mc13-f ACTGAATATAAACTTGTGGTAGTTGGCCCTGGT,

Mc13-r AGAAGCCTTTATGGCTATCAAAGAATGGTCCTGCACCAGTA.

PCR products for codons 12 and 13 were digested with restriction endonucleases BstNI or BglI (New England Biolabs, Ipswich, MA, USA), respectively, and analyzed on Experion Automated Electrophoresis Station (Bio-Rad, Hercules, CA, USA). For codon 12, cleavage of the 162 bp long PCR product yielded a 113 or 142 bp fragment, while for codon 13, the 174 bp product was truncated to a 165 or 125 bp fragment, depending on whether the allele was wild type or mutant, respectively.

### Statistical analyses

Data are expressed as mean±standard deviation (SD) if not stated otherwise. Student’s t-tests, ANOVA were used for comparison. A p-value of <0.05 was considered to be significant. All statistical analyses were performed by using the R-environment [24].

## Results

### Methylation profile of precursor lesions compared to CRC reveals more hypermethylated genes in precancerous lesions

Comparison of methylation profiles between CRCs, precancerous lesions (Table 1), inflamed and normal colorectal mucosa (Table 2) revealed that CRC and precancerous lesions have a characteristic methylation signature (Table 1). We identified a set of 10 genes that were hypermethylated in at least 85% of tumor samples (Table 1). These genes were not hypermethylated in MCRC, indicating that metastatic CRCs have a different epigenetic signature.

**Table 1:** Methylation status and frequency of top 10 hypermethylated genes in tumor samples. List of the top 10 genes that were hypermethylated in at least 85% of benign and malignant tumor samples. The highest mean methylation was observed in dysplastic lesions, followed by stage I-III cancer samples, whereas samples from patients with metastatic cancer had the lowest level of hypermethylation. SD: standard deviation. *Tumors: low-grade dysplasia, high-grade dysplasia, colorectal cancer stage I-III.

	Low-grade dysplasia (n = 17)		High-grade dysplasia (n = 6)		Colorectal cancer (n = 17)		Tumors* (n = 40)	Metastatic colorectal cancer (n = 7)	
Gene name	Mean methylation % ± SD	Methylated cases (%)	Mean methylation % ± SD	Methylated cases (%)	Mean methylation % ± SD	Methylated cases (%)	Methylated cases (%)	Mean methylation % ± SD	Methylated cases (%)
***SFRP1***	**60.66±25.27**	**100**	**54.93±30.99**	**100**	**31.67±16.36**	**94**	**97,5**	**7.56±2.1**	**0**
***SST***	**35.57±23.77**	**94**	**49.74±23.41**	**100**	**30.98±14.29**	**94**	**95**	**15.53±8.99**	**57**
***BNC1***	**63.95±24.45**	**100**	**53.8±17.43**	**100**	**31.99±14.53**	**88**	**95**	**20.15±3.63**	**57**
***MAL***	**51.79±30.94**	**100**	**41.39±30.32**	**83**	**37.14±18.83**	**88**	**92,5**	**10.21±9.15**	**14**
***SLIT2***	**58.58±32.95**	**100**	**47.79±29.48**	**100**	**26.32±17.86**	**82**	**92,5**	**9.12±3.4**	**0**
***SFRP2***	**48.49±34.63**	**82**	**51.63±30.57**	**100**	**27.49±14.35**	**94**	**90**	**5.26±4.52**	**0**
***SLIT3***	**38.44±30.9**	**94**	**41.46±22.63**	**100**	**27.08±21.36**	**76**	**87,5**	**5.78±8.52**	**14**
***ALDH1A3***	**53.95±38.12**	**88**	**74.36±18.83**	**100**	**37.22±23.74**	**76**	**85**	**6.73±2.64**	**0**
***TMEFF2***	**45.87±31.23**	**94**	**53.79±40.26**	**83**	**25.27±17.33**	**76**	**85**	**7.5±7.49**	**14**
***WIF1***	**44.27±30**	**88**	**49.89±40.45**	**83**	**30.32±21.16**	**82**	**85**	**19.1±16.62**	**29**

**Table 2:** Methylation status and frequency of the above top 10 hypermethylated genes in normal (young and adult), normal cancer adjacent (10 cm and 1 cm from the tumor margin) and ulcerative colitis (inactive and active) samples.

	Young normal (n = 5)		Adult normal (n = 5)		Cancer normal (10 cm, n = 5)		Field (1 cm, n = 5)		Inactive ulcerative colitis (n = 4)		Active ulcerative colitis (n = 4)	
Gene name	Mean methylation % ± SD	Methylated cases (%)	Mean methylation % ± SD	Methylated cases (%)	Mean methylation % ± SD	Methylated cases (%)	Mean methylation % ± SD	Methylated cases (%)	Mean methylation % ± SD	Methylated cases (%)	Mean methylation % ± SD	Methylated cases (%)
***SFRP1***	**0.52±0.36**	**0**	**4.95±4.69**	**0**	**4.95±4.62**	**0**	**3.12±2.48**	**0**	**8.16±12.89**	**25**	**3.23±2.91**	**0**
***SST***	**3.56±1.96**	**0**	**12.07±10.84**	**40**	**11.99±5.51**	**40**	**8.35±6.42**	**20**	**7.42±5.09**	**0**	**6.99±3.88**	**0**
***BNC1***	**2.02±1.0**	**0**	**16.37±17.49**	**40**	**11.67±8.14**	**20**	**8.71±5.11**	**20**	**12.84±10.01**	**25**	**7.83±3.86**	**0**
***MAL***	**1.03±0.49**	**0**	**2.65±3.28**	**0**	**3.71±4.06**	**0**	**1.75±0.83**	**0**	**1.38±0.99**	**0**	**1.49±0.63**	**0**
***SLIT2***	**1.68±0.89**	**0**	**2.58±2.0**	**0**	**6.93±8.21**	**20**	**4.05±1.89**	**0**	**6.85±6.02**	**25**	**4.06±1.87**	**0**
***SFRP2***	**1.79±1.26**	**0**	**1.55±0.99**	**0**	**2.33±2.39**	**0**	**2.27±0.64**	**0**	**2.51±1.82**	**0**	**2.5±0.72**	**0**
***SLIT3***	**0.61±0.3**	**0**	**2.49±2.81**	**0**	**1.36±1.02**	**0**	**1.25±0.43**	**0**	**4.93±5.52**	**0**	**1.39±0.56**	**0**
***ALDH1A3***	**0.75±0.45**	**0**	**4.12±3.32**	**0**	**3.77±1.9**	**0**	**2.82±1.69**	**0**	**3.61±2.74**	**0**	**2.62±1.19**	**0**
***TMEFF2***	**1.36±0.71**	**0**	**1.23±1.07**	**0**	**1.93±1.69**	**0**	**1.46±1.05**	**0**	**2.73±4.09**	**0**	**1.94±0.63**	**0**
***WIF1***	**2.63±1.62**	**0**	**8.14±6.78**	**20**	**13.75±8.8**	**20**	**8.59±5.34**	**0**	**16.22±20.39**	**20**	**4.61±3.89**	**0**

The highest mean methylation in the tumor samples were observed in HGD, followed by LGD, then by CRC, being the lowest in MCRC (Table 3).

**Table 3:** Mean DNA methylation levels for hypermethylated genes sorted by group.

A.	Y	N	CN (10 cm)	F (1 cm)	LGD	HGD	CRC	MCRC	UCi	UCa
***BAGE***	**97.39±1.32**	**76±15.83**	**79.72±11.97**	**66.2±21.11**	**95.43±3.35**	**93.46±8.09**	**82.52±18.2**	**90±11.83**	**76.65±5.61**	**78.4±7.83**
***CCNA1***	2.75±1.48	**30.76±23.14**	**24.74±10.49**	**18.17±10.12**	**67.1±23.44**	**61.99±17.72**	**43.94±9.66**	**33.46±9.6**	**23.7±20.65**	**8.96±4.12**
***H19***	**47.91±2.89**	**46.33±5.82**	**37.36±7.5**	**37.17±11.42**	**33.95±13.46**	**38.72±15.54**	**44.16±17.53**	**42.35±4.89**	**40.96±12.74**	**46.39±6.27**
***MAGEA1***	**98.18±1.14**	**73.73±13.92**	**86.44±9.69**	**90.08±4.23**	**79.14±33.83**	**61.85±44.1**	**76.63±25–96**	**90.23±13.21**	**63.94±15.08**	**80.93±7.84**
***MSX1***	**13.9±3.3**	**17.26±3.96**	**20.2±10.87**	**15.66±5.46**	**66.64±24.29**	**57.98±14.36**	**46.63±17.95**	**29.4±7.3**	**34.95±17.57**	**20.57±4.08**
***PTGIS***	**22.95±13.09**	**43.84±10.57**	**49.64±4.72**	**43.29±10.54**	**79.98±17.71**	**79.14±17.34**	**59.71±15.84**	**56.29±18.53**	**44.67±20.47**	**39.43±11.62**
***RUNX3***	**34.95±11.62**	**42.96±12.45**	**38.94±7.53**	**37.9±10.99**	**71.33±21.56**	**69.82±12.09**	**58.52±13.82**	**52.39±9.39**	**41.59±13.79**	**56.96±5.15**
***SPARC***	**21.42±8.49**	**42.08±10.87**	**42.86±6.75**	**38.97±11.28**	**65.02±19.86**	**49.02±33.01**	**46.86±15.24**	**45.43±6.01**	**31.75±17.67**	**31.34±15.04**
***UGT1A1***	**84.15±6.49**	**51.13±3.28**	**86.53±8.32**	**85.75±5.88**	**91.49±9.46**	**89.29±10.02**	**94.9±4.69**	**81.26±22.83**	**47.49±3.42**	**58.42±3.94**
**B.**										
***BNC1***	2.02±1.0	16.37±17.49	11.67±8.14	8.71±5.11	**63.95±24.45**	**53.8±17.43**	**31.99±14.53**	**20.15±3.63**	12.84±10.01	7.83±3.86
***SST***	3.56±1.96	12.07±10.84	11.99±5.51	8.35±6.42	**35.57±23.77**	**49.74±23.41**	**30.98±14.29**	**15.53±8.99**	7.42±5.09	6.99±3.88
**C.**										
***ADAMTS1***	0.56±0.66	0.37±0.34	0.49±0.75	0.52±0.39	**19.38±29.92**	**43.26±35.45**	**18.18±19.41**	1.23±0.87	0.83±0.57	0.15±0.57
***ALDH1A3***	0.75±0.45	4.12±3.32	3.77±1.9	2.82±1.69	**53.95±38.12**	**74.36±18.83**	**37.22±23.74**	6.73±2.64	3.61±2.74	2.62±1.19
***ALX4***	0.72±0.59	0.69±0.57	0.8±0.84	0.72±0.2	**33.94±35.79**	**51.47±41.78**	**27.76±26.83**	1.26±1.04	1.34±0.98	0.94±0.42
***CDH13***	1.6±0.92	1.23±0.85	3.93±3.82	2.47±0.98	**32.79±34.53**	**33.47±27.99**	**25.75±25.35**	3.7±2.82	2.42±2.64	3.08±1.56
***DKK2***	4.21±0.93	8.6±4.47	5.09±3.25	4.13±0.99	**39.93±25.97**	**46.7±17.31**	**31.54±18.05**	7.04±2.99	11.13±13.86	9.11±4.77
***DKK3***	1.61±1.45	1.95±0.52	2.02±2.76	1.31±0.19	**21.04±28.46**	**32.3±32.37**	**23.36±19.79**	4.76±5.03	2.89±2.56	1.9±1.03
***GALR2***	2.24±1.02	2.2±1.65	3.71±2.38	2.18±0.51	**37.63±33.64**	**50.16±29.88**	**25.88±24.27**	5.79±4.12	4.6±2.73	4.33±2.42
***HLTF***	5.06±1.44	5.39±1.79	4.45±1.34	4.78±1.51	**19.71±15.27**	**33.01±27.9**	**18.36±15.74**	8.17±4.72	6.35±7.34	8.91±4.6
***hsa-mir-342***	0.53±0.5	1.18±1.61	2.82±2.98	2.14±3.09	**42.73±22.86**	**17.71±27.98**	**21.27±16.69**	3.81±3.23	0.39±0.33	0.4±0.27
***LRRC3B***	1.44±0.93	3.59±3.61	1.96±1.2	1.1±0.66	**26.39±12**	**35.55±24.78**	**12.68±10.04**	4.94±1.81	3.1±2.77	2.14±1.34
***MAL***	1.03±0.49	2.65±3.28	3.71±4.06	1.75±0.83	**51.79±30.94**	**41.39±30.32**	**37.14±18.83**	10.21±9.15	1.38±0.99	1.49±0.63
***NID1***	1.26±0.83	1.43±0.64	1.52±1.52	1.13±0.23	**26.86±29.06**	**24.35±29.84**	**19.33±19.46**	2.28±2.12	2.55±1.6	1.78±0,92
***OPCML***	2.49±1.05	9.96±11.52	13.03±11.34	7.13±3.54	**47.01±19.98**	**38.1±12.77**	**20.62±15.2**	12.54±3.24	8.74±10.68	4.79±1.95
***PAX2***	1.16±0.5	0.81±0.65	1.19±1.04	1.26±1.02	**26.5±34.37**	**42.74±37.46**	**19.97±19.1**	1.23±1.33	1.49±1.73	0.99±0.53
***PCDH10***	1.53±0.52	3.44±1.4	3.98±1.89	3.4±1.24	**44.79±29.1**	**57.28±35.04**	**21.77±18.49**	7.43±5.2	7.73±10.11	3.65±1.8
***PDLIM4***	2.75±0.99	12.22±11.22	7.14±3.09	7.48±4.1	**40.94±39.99**	**77.39±20.48**	**28.69±21.67**	12.48±7.97	13.24±8.87	7.05±3.16
***RBP1***	1.18±0.52	1.44±0.81	3.08±2.29	1.15±0.45	**42.22±39.03**	**39.55±38**	**20.34±13.8**	3.88±4.7	1.57±1.79	1.9±0.86
***RPRM***	2.19±0.95	2.97±1.62	4.38±1.87	2.81±0.66	**25.62±28.93**	**26.88±33**	**21.41±19.23**	10.25±6.48	8.4±6.9	4.76±1.26
***SFRP1***	0.52±0.36	4.95±4.69	4.95±4.62	3.12±2.48	**60.66±25.27**	**54.93±30.99**	**31.67±16.36**	7.56±2.1	8.16±12.89	3.23±2.91
***SFRP2***	1.79±1.26	1.55±0.99	2.33±2.39	2.27±0.64	**48.49±34.63**	**51.63±30.57**	**27.49±14.35**	5.26±4.52	2.51±1.82	2.5±0.72
***SLC16A12***	0.85±0.94	0.89±0.63	1.64±1.67	0.92±0.23	**25.78±29.43**	**41.5±39.64**	**21.53±21.37**	2.24±1.3	4.04±4.44	1.76±0.94
***SLIT2***	1.68±0.89	2.58±2	6.93±8.21	4.05±1.89	**58.58±32.95**	**47.79±29.48**	**26.32±17.86**	9.12±3.4	6.85±6.02	4.06±1.87
***SLIT3***	0.61±0.3	2.49±2.81	1.36±1.02	1.25±0.43	**38.44±30.9**	**41.46±22.63**	**27.08±21.36**	5.78±8.52	4.93±5.52	1.39±0.56
***TAC1***	2.2±1.63	6±4.42	4.73±2.38	8.9±8.73	**22.51±27.98**	**30.17±16.38**	**18.84±19.05**	15.36±10.11	15.39±14.47	6.13±2.19
***TMEFF2***	1.36±0.71	1.23±1.07	1.93±1.69	1.46±1.05	**45.87±31.23**	**53.79±40.26**	**25.27±17.33**	7.5±7.49	2.73±4.09	1.94±0.63
***UCHL1***	2.83±1.21	2.68±2.3	2.07±2.23	2.43±0.04	**29.26±35**	**57.78±34.35**	**23.29±17.5**	6.27±6.03	2.24±1.62	3.79±1.9
***VIM***	1.54±0.65	0.65±0.5	0.89±0.99	0.84±0.88	**40.03±34.38**	**46.53±40.81**	**27.35±22.14**	3.32±3.86	2±1.63	1.92±1.27
***WIF1***	2.63±1.62	8.14±6.78	13.75±8.8	8.59±5.34	**44.27±30**	**49.89±40.45**	**30.32±21.16**	19.1±16.62	16.22±20.39	4.61±3.89
**D.**										
***DAB2IP***	1.12±0.35	1.35±0.18	0.8±0.4	0.59±0.21	**20.01±26.99**	**27.52±23.65**	8.7±7.24	6.33±10.34	2.81±0.49	0.73±0.25
***HS3ST2***	1.35±0.6	1.36±0.12	1.16±1.02	0.81±0.03	**31.13±36.33**	**29.69±26.42**	13.45±12.23	1.88±1.55	3.28±1.01	0.78±0.16
***IGFBP3***	0.23±0.32	0.22±0.26	0.3±0.29	0.29±0.21	**11.03±15.37**	**22.92±30.86**	11.98±18.37	0.5±0.5	1±1.18	0.47±0.2
***WT1***	0.44±0.25	0.73±0.56	0.97±1.07	0.4±0.34	**22.06±31.12**	**31.78±31.99**	12.99±19.58	1.18±1.15	1.78±1.86	0.85±0.84
***CALCA***	8.37±1.09	8.78±2.71	8.22±4.01	13.57±11.74	8.72±4.24	**30.74±27.13**	**18.92±14.32**	12.29±5.2	14.52±9.31	18.07±3.38
***MCC***	4.74±1.71	2.78±1.19	2.89±1.87	2.12±0.93	11.99±14.96	**34.09±28.85**	**17.38±15.11**	6.87±6.61	3.61±2.91	5.87±3.14
***BMP3***	0.17±0.23	0.12±0.02	0.18±0.26	0.07±0.02	8.65±18.27	**31.09±30.12**	9.46±13.85	0.21±0.27	0.27±0.07	0.07±0.01
***CHFR***	0.46±0.09	0.41±0.23	0.3±0.16	0.35±0.08	6.83±11.39	**20.56±27.57**	5.4±6.1	0.25±0.53	0.42±0.74	0.28±0.18
***DACT2***	0.67±0.17	0.75±0.39	1.26±1.02	1.17±0.6	4.23±6.02	**33.62±31.64**	6.41±11.25	1.82±1.69	2.14±1.89	2.3±0.64
***EYA2***	2.06±0.96	0.55±0.5	1.16±1.46	1±0.27	3.6±5.86	**30.98±37.86**	10.84±16.92	1.4±1.37	2.24±1.47	2.07±1
***NKX2***	0.78±0.62	0.88±0.48	0.68±0.72	0.55±0.12	6.82±8.83	**34.85±41.31**	6.17±10.95	1.26±0.67	1.83±0.56	1.2±0.42
***SFRP5***	2.51±1.58	2.61±1.93	1.12±1.21	0.95±0.36	15.57±27.23	**15.35±10.84**	9.24±10.4	2.09±1.36	2.95±0.8	3.3±1.86
***SLC5A8***	0.85±0.44	3.42±3.73	2.69±1.75	3.58±1.29	12.15±13.15	**34.12±42.53**	11.46±19.21	4.59±3.11	2.24±0.81	3.52±1.01
***TFAP2C***	2.43±1.06	0.99±1.19	1.1±1.37	0.63±0.42	12.47±19.71	**26.11±38.62**	7.27±15.74	1.78±2.38	1.9±0.9	3.16±1.47
***WNT5A***	0.37±0.38	0.05±0.07	0.03±0.03	0±0	0.75±2.19	**21.44±31.86**	0.8±2.17	0.18±0.35	0.17±0.32	0.05±0.1
***WRN***	1.75±0.79	1.51±0.16	3.55±1.01	3.68±1.03	2.82±1.89	**19.17±16.27**	5.7±7.5	2.41±1.22	3.73±1.14	1.24±0.59

**A.** Genes commonly methylated in >95% of all samples.

**B.** Genes methylated in all tumorous samples (including MCRC).

**C.** Genes methylated only in LGD, HGD and stage I-III CRC.

**D.** Genes individually methylated in certain groups.

Y: young normal, N: adult normal, CN: macroscopically normal tissue taken from at least 10 cm away from the tumor margin, F: macroscopically normal tissue taken from 1 cm away from the tumor margin, LGD: low-grade dysplasia, HGD: high-grade dysplasia, CRC: colorectal cancer, MCRC: metastatic CRC, UCi: inactive ulcerative colitis, UCa: active ulcerative colitis. Bold: genes hypermethylated in more than half of the samples in the representative group.

DNA methylation profiling revealed an increasing number of methylated genes along ACS. In addition to the 9 hypermethylated genes found in the majority (>95%) of samples, including normal and UC tissue (Table 3), additional 34, 46 and 32 hypermethylated genes were observed in more than half of LGD, HGD and CRC samples, respectively. In MCRC only 2 additional hypermethylated genes were observed (Table 3). A set of 12 genes were exclusively hypermethylated in HGD (Table 3). The frequency of hypermethylated DNA copies was also significantly higher in the premalignant alterations than in cancer.

### Methylation levels and penetrance of genes in precancerous and CRC lesions: confirmation from parallel alterations

**Fig 1:**
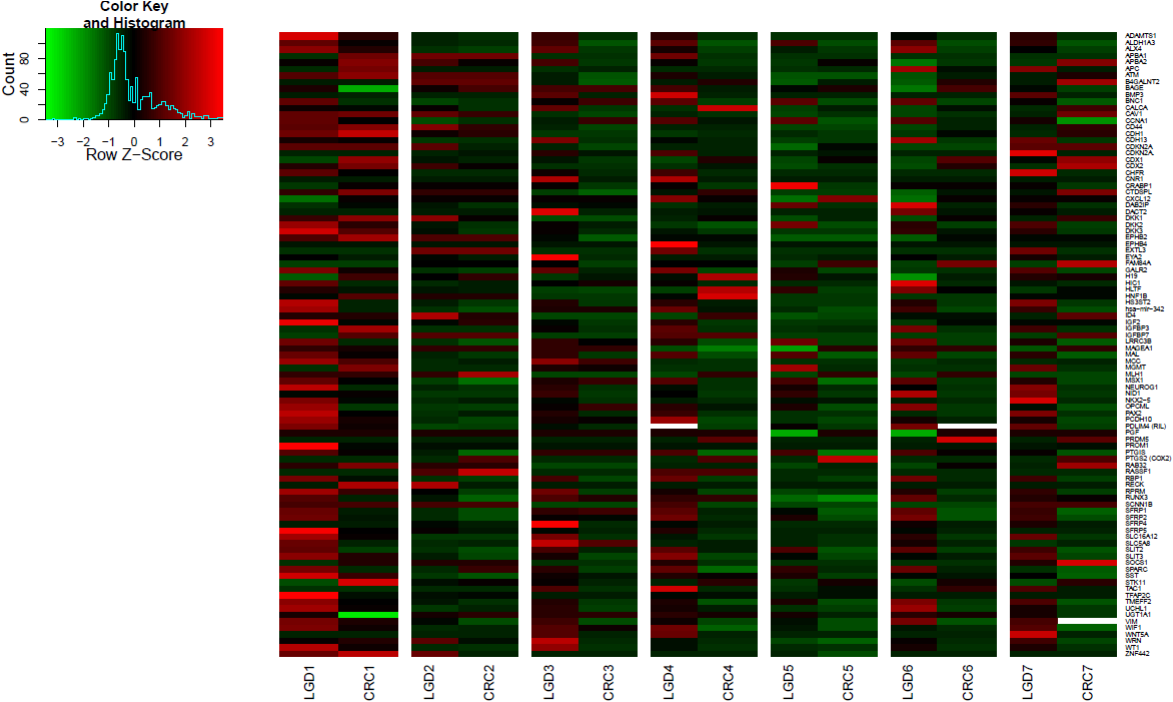
DNA methylation heatmap for synchronous LGD and CRC samples from the same patient. The number of hypermethylated genes and methylation levels were higher in LGD than in synchronous CRC.

The level of DNA methylation was higher in precancerous lesions (LGD and HGD), compared to CRC (Table 3). This observation was further validated in 7 synchronous LGD-CRC pairs where methylation levels proved to be greater in LGD than in synchronous CRC (Fig 1, S2 Fig). This observation was also confirmed by bisulfite-HRM analysis in 3 genes and showed consistent results (S3 Fig). The number of hypermethylated genes was also higher in LGD, than in synchronous CRC (Fig 1, S2 Fig). The mean DNA methylation percentage of methylated genes was significantly higher in LGD than in CRC (S2 Fig). The comparison of DNA methylation profiles between CRC and NAT revealed that hypermethylation occurred only in cancer but not in the adjacent field (Table 3A–3D).

### Hypermethylated genes have a higher penetrance than mutations

All samples were MSS. 25% of the samples harbored *KRAS* mutations and 10% exhibited *BRAF* V600E mutation (Table 4). Although only 2 genes merely does not represent all genetic mutations, but even these frequent mutations occur at a much lower frequency than hypermethylated genes, which indicates that hypermethylated genes have a higher penetrance than mutations, but this should be further evaluated studying more mutations.

**Table 4:** Frequency and percentage of *KRAS* and *BRAF* mutations in normal (N), low-grade dysplasia (LGD), high-grade dysplasia (HGD) and colorectal cancer (CRC) samples.

	n	*KRAS* c12	%	*KRAS* c13	%	*BRAF* V600E	%
**N**	20	0	0	0	0	0	0
**LGD**	17	4	24%	0	0	3	18%
**HGD**	6	1	17%	0	0	1	17%
**CRC**	17	4	24%	1	6%	0	0
**Total**	**40**	**9**	**22.5%**	**1**	**2.5%**	**4**	**10%**

### Active and passive long-term chronic inflammation resemble the normal pattern

**Fig 2:**
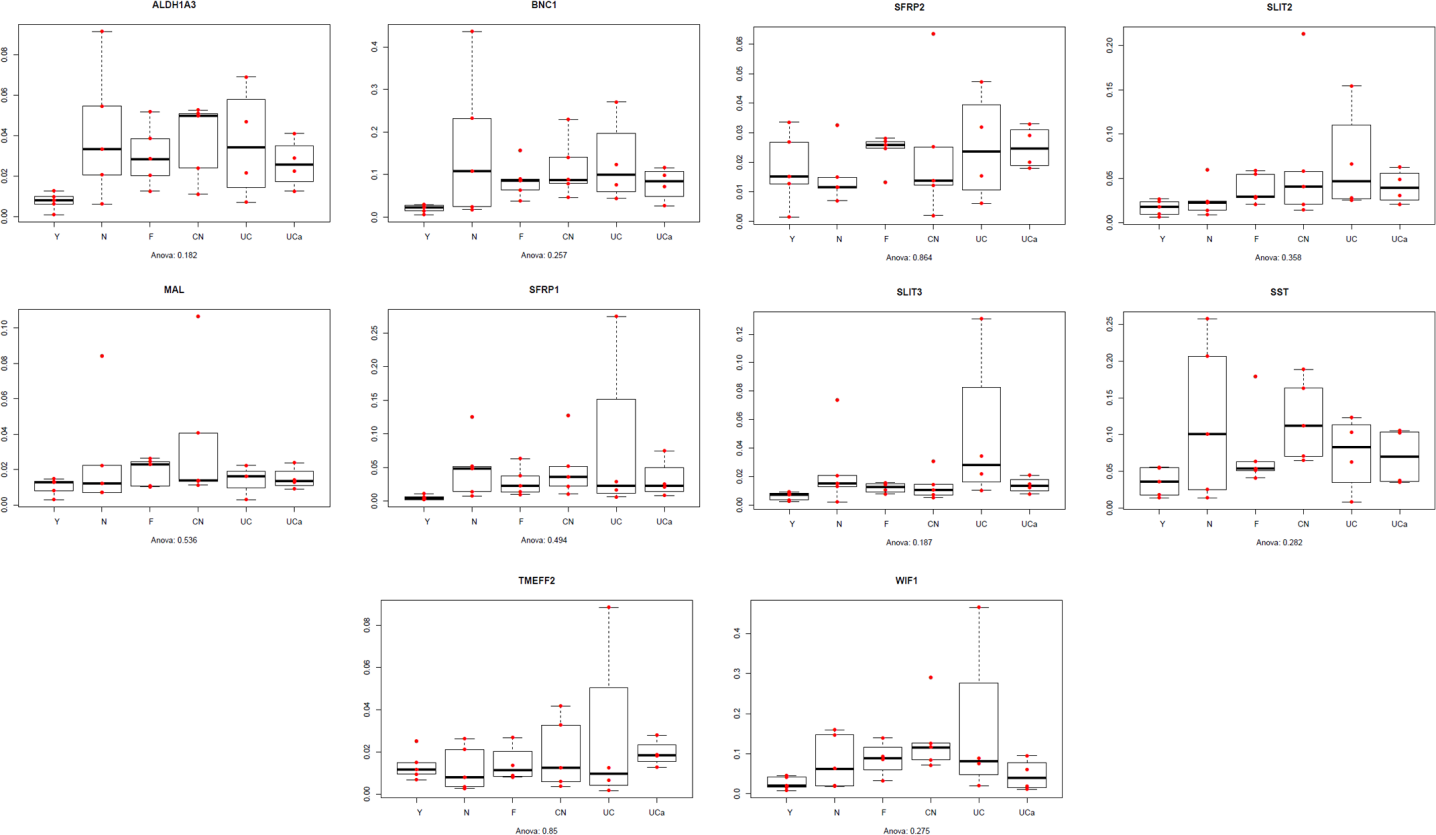
Methylation levels in various groups in the top 10 hypermethylated genes. No significant methylation changes were observed among different groups. Y: young normal, N: adult normal, CN: macroscopically normal tissue taken from at least 10 cm away from the tumor margin, F: macroscopically normal tissue taken from 1 cm away from the tumor margin, LGD: low-grade dysplasia, HGD: high-grade dysplasia, CRC: colorectal cancer, MCRC: metastatic CRC, UC: inactive ulcerative colitis, UCa: active ulcerative colitis

We analyzed the methylation profile of 4 samples with UCa and 4 samples with UCi. Although we found difference between methylation levels (e.g. 63% vs 80%, Table 3), both conditions showed a similar pattern to that of normal samples, no significant changes were observed and no additional gene was identified as hypermethylated (Fig 2).

### Correlation between DNA methylation, mRNA and protein expression

**Fig 3:**
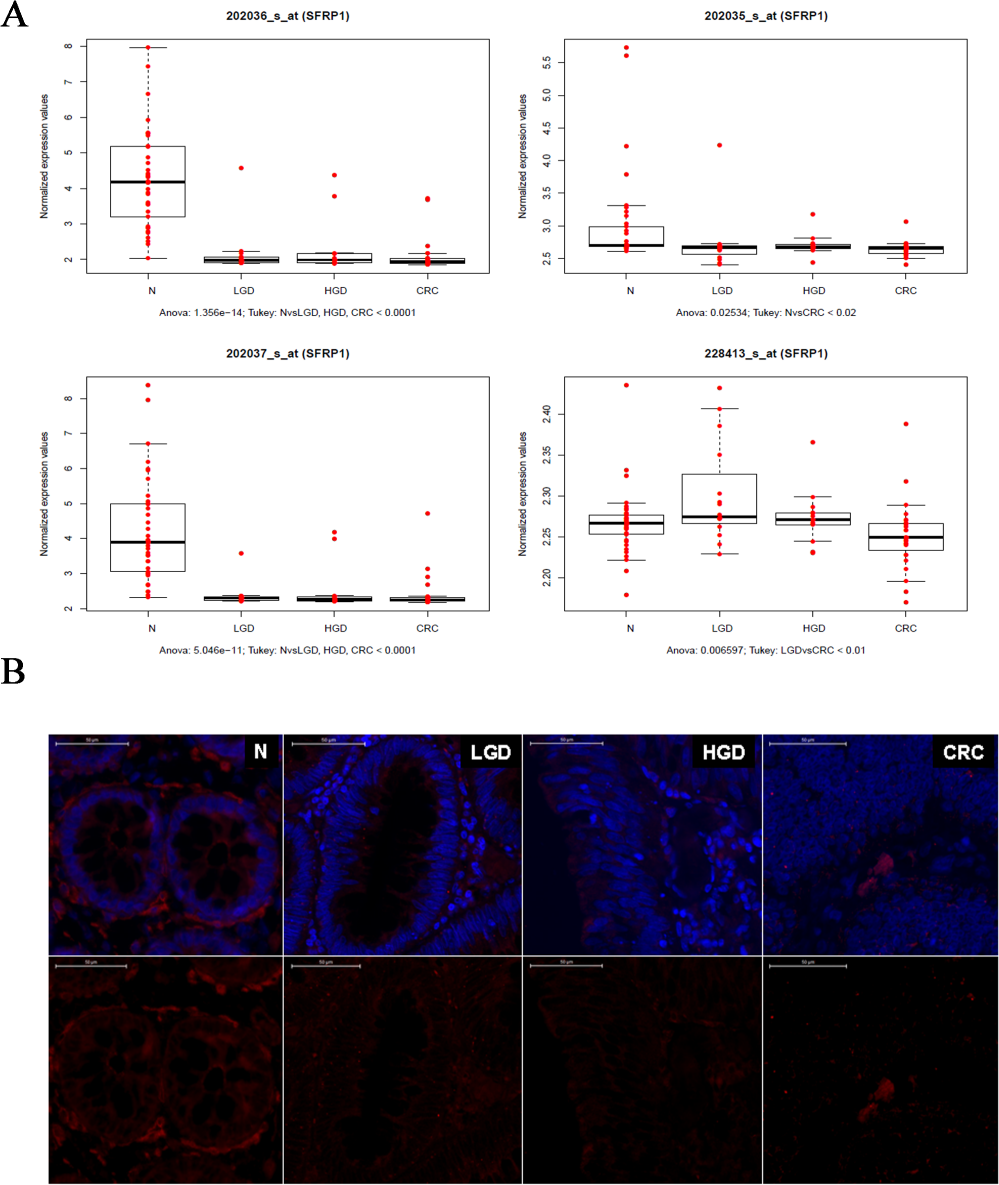
A. Gene expression of *SFRP1* during ACS measured by mRNA expression microarray analysis. As indicated, transcript levels decreased across in LGD, HGD and CRC samples in 3 out of 4 *SFRP1* transcripts. B. Protein expression of SFRP1 protein measured by immunohistochemistry. SFRP1 protein expression gradually decreased during ACS. The epithelial SFRP1 expression showed a continuous decrease during ACS. Epithelial SFRP1 expression was decreased in LGD compared to normal epithelium, but stromal SFRP1 protein expression was retained. In HGD and CRC samples, both epithelial and stromal SFRP1 protein expression was significantly reduced. Red staining: SFRP1, blue staining: Hoechst nuclear staining. N: normal sample, LGD: low-grade dysplasia, HGD: high-grade dysplasia, CRC: colorectal cancer

To analyze the effect of DNA hypermethylation, we examined mRNA and protein expression levels of *SFRP1*, a well-described antagonist of the Wnt pathway, frequently aberrantly activated in CRC. We confirmed the findings of previous studies, that DNA hypermethylation leads to subsequent underexpression of SFRP1 mRNA (Fig 3A) and lower protein levels (Fig 3B) in CRC, and also in precursor lesions. In normal samples we found strong, diffuse cytoplasmic SFRP1 protein expression in the epithelium and in the stroma, as well. In the stroma, SFRP1 positive cells were localized primarily in close proximity to epithelial cells. In LGD samples, epithelial SFRP1 protein expression decreased, but in the stroma, it was similar to that observed in normal samples. In HGD, reduction in epithelial SFRP1 protein expression was more pronounced than in LGD. In both HGD and CRC, there was no apparent stromal expression of SFRP1 proximal to the epithelia.

### Demethylation treatment increases mRNA expression level of hypermethylated genes

**Fig 4:**
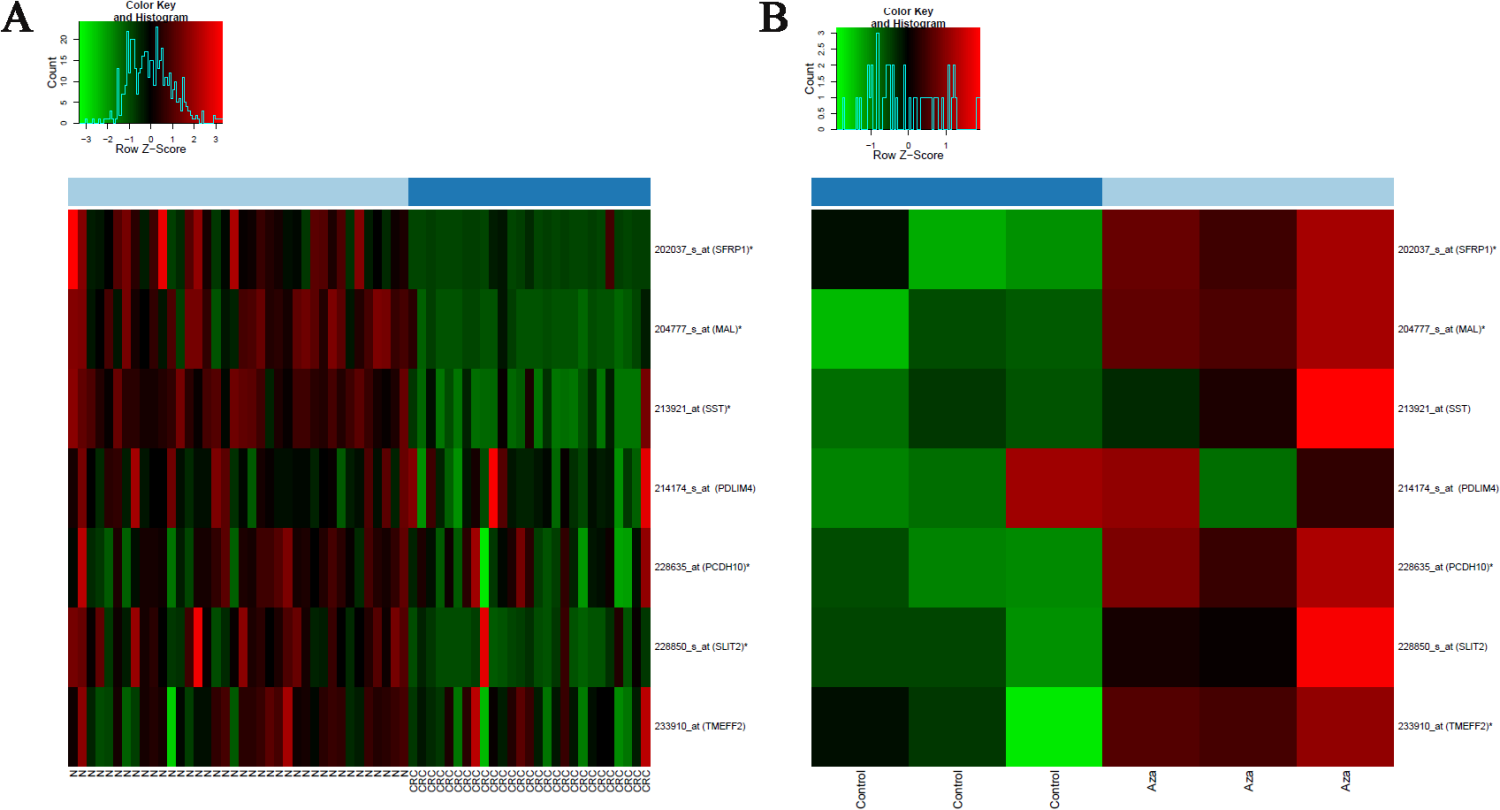
mRNA expression of 7 genes in normal and colorectal cancer biopsy (A) and in HT29 cells after 5-aza-2’-deoxycytidine treatment (B). **A.** Compared to normal, CRC showed decreased mRNA expression. **B.** In HT29 cells, demethylation treatment partly restored mRNA expression. N: normal, CRC: colorectal cancer, AZA: 5-aza-2’-deoxycytidine treatment.

Whole genomic mRNA expression microarray data was utilized to analyze mRNA expression of 7 genes previously identified as hypermethylated in CRC. These genes showed decreased mRNA expression in CRC compared to normal tissue in the majority of the examined samples (Fig 4A).

To examine whether downregulation of these gene can be reversed by demethylation, 5-aza-2’-deoxycytidine treatment was applied to HT-29 cells. The treatment was carried out in 3 parallel experiments and partly restored mRNA expression of these genes, indicating that DNA hypermethylation seems to play a role in the regulation of these genes (Fig 4B). Similar tendency was oberved in another experiment, where 5-aza-2’-deoxycytidine treatment was applied to HCT116 cell line (S4 Fig).

## Discussion

In this study we compared DNA methylation profiles of normal colorectal tissue, colorectal precancerous lesions, and CRC tissue. We also examined the effect of chronic inflammation and field effect. We focused on distal colorectal carcinogenesis, which is known to emerge via the traditional adenoma-cancer pathway and driven by the sequentional accumulation of genetic mutations. In a recent genome-wide array-based study 2 classes (high-frequency methylation and low-frequency methylation) of adenomas were identified based on their DNA methylation patterns [25]. We only included MSS samples to ensure molecular homogeneity in our study. To date, there has only been one study dedicated to the characterization of DNA methylation in CIMP-negative tumors [26]. This study however focused only on CRC, examined few genes, and did not characterize precursor lesions. Furthermore, we used a wider set of 96 candidate genes, for which hypermethylation has frequently been described in gastrointestinal cancers.

Our results indicate that there is a characteristic methylation pattern for MSS CRCs, but also for their precursor lesions. We identified a set of 10 genes that are frequently hypermethylated in benign and malignant colorectal tumors. More interestingly precursor lesions had more hypermethylated genes and a higher grade of methylation than CRC, when synchronous LGD and CRC pairs were compared. One possible explanation for this phenomenon would be the higher epithelium-stroma ratio in hyperprolipherative precursor lesions, than in CRC. According to an other theory, since cancerous cells develop from dysplastic cells, CRC samples could contain significant amount of dysplastic (but not yet cancerous) cells. In that case the hypermethylation of 10 genes can be specific for dysplastic samples and their presence in CRC samples is a result of “contamination” with dysplastic cells. Further characterization of the 10 genes did not reveal common features as they were located on 7 different chromosomes and have distinct biological functions. The examined genes were seperately evaluated and characterized for CRC in previous studies [27–43]. Interestingly, in most studies these markers showed a similar or somewhat weaker performance, as compared to our results (Table 5). We have to note that our study used fresh frozen samples and a real time PCR restriction-based method. These differences in methodology may contribute to the better sensitivity as compared to those observed in previous studies. Furthermore, we confirmed that metastatic CRCs have a different DNA methylation fingerprint [44], as most of the genes hypermethylated in precancerous and cancerous lesions were not methylated in MCRC. We think that hypermethylated genes characteristic for MCRC might have not been included in our array, however this should be further evaluated in subsequent studies.

**Table 5:** Comparison of performance of top genes in our study and previous single gene studies.

	N (%)	LGD (%)	HGD (%)	CRC (%)	TU (%, n = 40)	Reference	N	%	AD	%	CRC	%	Method
***SFRP1***	**0**	**100**	**100**	**94**	**97,5**	Caldwell 2004	**11/36**	**30%**	**n.a.**	** **	**40/49**	**82%**	**MSP, COBRA**
						Qi 2006	**0/20**	**0%**	**29/33**	**88%**	**67/72**	**88%**	**MSP**
***SST***	**18**	**94**	**100**	**94**	**95**	Mori 2006	**n.a.**		**n.a.**	** **	**30/34**	**88%**	**MSP**
***BNC1***	**18**	**100**	**100**	**88**	**95**	Shames 2006	**n.a.**		**10/24**	**42%**	**22/24**	**92%**	**MSP**
***MAL***	**0**	**100**	**83**	**88**	**92,5**	Lind 2008	**1/23**	**4%**	**45/63**	**71%**	**49/61**	**80%**	**MSP**
***SLIT2***	**7**	**100**	**100**	**82**	**92,5**	Dallol 2003	**n.a.**		**n.a.**		**23/32**	**72%**	**BS**
***SFRP2***	**0**	**82**	**100**	**94**	**90**	Qi 2006	**0/20**	**0%**	**27/33**	**82%**	**60/72**	**83%**	**MSP**
***SLIT3***	**0**	**94**	**100**	**76**	**87,5**	Dickinson 2004	**n.a.**		**n.a.**		**12/32**	**38%**	**COBRA**
***ALDH1A3***	**0**	**88**	**100**	**76**	**85**	Shames 2006	**n.a.**	** **	**7/17**	**29%**	**11/24**	**46%**	**MSP**
***TMEFF2***	**0**	**94**	**83**	**76**	**85**	Ebert 2005	**1/21**	**5%**	**n.a.**	** **	**36/47**	**71%**	**Methylight**
***WIF1***	**11**	**88**	**83**	**82**	**85**	Taniguchi 2005	**n.a.**	** **	**32/44**	**73%**	**41/50**	**82%**	**MSP**
***PCDH10***	**4**	**88**	**83**	**76**	**82,5**	Zhong 2013	**n.a.**	** **	**n.a.**	** **	**68/80**	**85%**	**MSP**
***PDLIM4 (RIL)***	**11**	**82**	**100**	**76**	**82,5**	Boumber 2007	**1/22**	**5%**	**11/13**	**85%**	**30/43**	**70%**	**COBRA**
***VIM***	**0**	**82**	**83**	**76**	**80**	Kann 2006	**0/11**	**0%**	**25/50**	**50%**	**72/94**	**77%**	**MSP**
***GALR2***	**0**	**76**	**100**	**71**	**77,5**	Chung 2008	**0/20**	**0%**	**n.a.**	** **	**17/20**	**85%**	**COBRA, PS**
***DKK2***	**7**	**88**	**100**	**59**	**77,5**	Sato 2007	**n.a.**	** **	**n.a.**	** **	**45/58**	**78%**	**MSP**
***OPCML***	**14**	**94**	**83**	**59**	**77,5**	Cui 2008	**n.a.**	** **	**n.a.**	** **	**17/18**	**94%**	**MSP**
***UCHL1***	**0**	**65**	**83**	**82**	**75**	Okochi-Takada 2006	**1/17**	**6%**	**n.a.**	** **	**8/17**	**47%**	**MSP**
						Mizukami 2008	**n.a.**		**n.a.**		**36/49**	**73%**	**MSP**

COBRA: Combined bisulfite restriction analysis, MSP: methylation-specific polymerase chain reaction, PS: pyrosequencing, N: normal, LGD: low-grade dysplasia, HGD: high-grade dysplasia, CRC: colorectal cancer, TU: precancerous and cancerous tissue (except metastatic colorectal cancer), AD: adenoma (level of dysplasia not specified).

We showed that 7 genes had decreased mRNA expression in tumorous samples, and this decreased mRNA expression could be partly restored by demethylation treatment. This indicates that DNA hypermethylation might play a role in the regulation of these genes and demethlyation could potentially reverse these changes indicating a yet undiscovered systematic underlying mechanism, however this should be further studied to draw firm conclusions. Among these, *SFRP1* was analyzed also at the protein level and showed decreased protein expression compared to healthy samples. Concerning SFRP1 our data are consistent with published studies [27,45], which provide evidence for hypermethylation and consequential underexpression of SFRP1, promoting tumor formation. SFRPs are well-known inhibitors of the Wnt pathway. Abnormal activation of the Wnt pathway (e.g. via APC mutation, beta-catenin translocation) is a frequent and early event in colorectal carcinogenesis [4]. The hypermethylation of this gene may also be an early event that can be harnessed in future studies addressing the potential of hypermethylated SFRP1 in early detection of CRC. As we along with others have shown, DNA methylation is a promising blood-based biomarker for early detection of CRC and could potentially substitute routinely used, more invasive screening methods [46–48].

When the frequency of mutations and hypermethylated genes in tumorous samples was compared, methylated genes showed a significantly higher penetrance than mutations. We identified 10 genes that were hypermethylated in more than 85% of the examined samples, whereas mutations were less frequent, further underscoring the importance of DNA methylation in colorectal carcinogenesis.

One aspect of this study was to determine if we could correlate changes in DNA methylation with initial steps in the carcinogenesis pathway, first in terms of field effect, and secondly in chronic inflammation, a condition considered as a CRC risk factor. With respect to field effect, Shen *et al* observed the DNA repair gene O^6^-methylguanine-DNA methyltransferase (*MGMT*) to be hypermethylated and silenced in colorectal tumors, but also in the surrounding mucosa [49], providing proof of principle for the field effect of methylation in CRC. This phenomenon was later described for several other genes [50]. Our samples were taken 1 cm and 10 cm away from the margin of the tumor, from the macroscopically normal colorectal mucosa. These showed a similar pattern to healthy controls, suggesting the lack of a field effect for genes in this study. We have to note, that all of our samples were MSS, whereas methylation of *MGMT* was associated with the development of low level microsatellite instability (MSI-L) CRC [51].

With respect to chronic inflammation, this condition has been proposed to lead to DNA hypermethylation in non-cancerous tissues, that can be detected and used as a risk factor for cancer [8]. However, our study did not reveal any DNA methylation markers that were predictive of CRC in ulcerative colitis patients as a model for chronic inflammation of the gut.

In conclusion, our study has confirmed that hypermethylation of certain genes is characteristic for MSS cancer. We have shown for the first time that precursor lesions, especially those with low-grade dysplasia, exhibit more hypermethylated genes. These genes are more densely hypermethylated, than their matched cancer pairs. We identified potential hypermethylated genes, including *SFRP1* and *MAL* that might be useful in the early detection of CRC and help to prevent this otherwise deadly disease.

## Supporting Information

S1 Fig Treshold for hypermethylation at different genes. Threshold for hypermethylation was set at 15% after comparing normal and cancerous samples in several genes.
(PDF)

S2 Fig Mean DNA methylation percentage of methylated genes in synchronous LGD and CRC. The mean DNA methylation percentage of methylated genes was significantly higher in LGD than in CRC. LGD: low-grade dysplasia, CRC: colorectal cancer
(TIF)

S3 Fig High resolution melting (HRM) profile derivative plots (-d/dT against T). The inflection point on each standard melting curve is visualized here as a melting peak. Panels on the left show the degree of DNA methylation in normal (N) versus colorectal carcinoma (CRC) and panels on the right show that in low-grade dysplasia (LGD) versus CRC tissues. Sample pairs were obtained from the same patient. The melting peaks of methylated DNA standards (0% and 100%) and no template control (NTC) are also indicated for each gene studied.
(TIF)

S4 Fig Effect of demethylation (5-aza-2’-deoxycytidine) treatment on HCT116 cell line from Yagi et al [20]. mRNA expression of some genes can be partly reversed by 5-aza-2’-deoxycytidine and trichostatin-A treatment on HCT116 cell line. HCT116 aza0: untreated, HCT116 aza3: 3 μM 5-AZA treatment for 72 hours, HCT116 aza3_tsa300: additional trichostatin-A (HDAC inhibitor) treatment. White squares indicate that gene expression was unchanged in that experiment.
(PDF)

S1 Table The list of examined genes on Methyl Profiler microarray. (DOCX)
