# Supplementary figures and images for: Comprehensive DNA Methylation Analysis Reveals a Common Ten-Gene Methylation Signature in Colorectal Adenomas and Carcinomas
Source: PLoS One. 2015 Aug 20;10(8):e0133836. doi: 10.1371/journal.pone.0133836 (PMC4546193; doi:10.1371/journal.pone.0133836)

**ALDH1A3**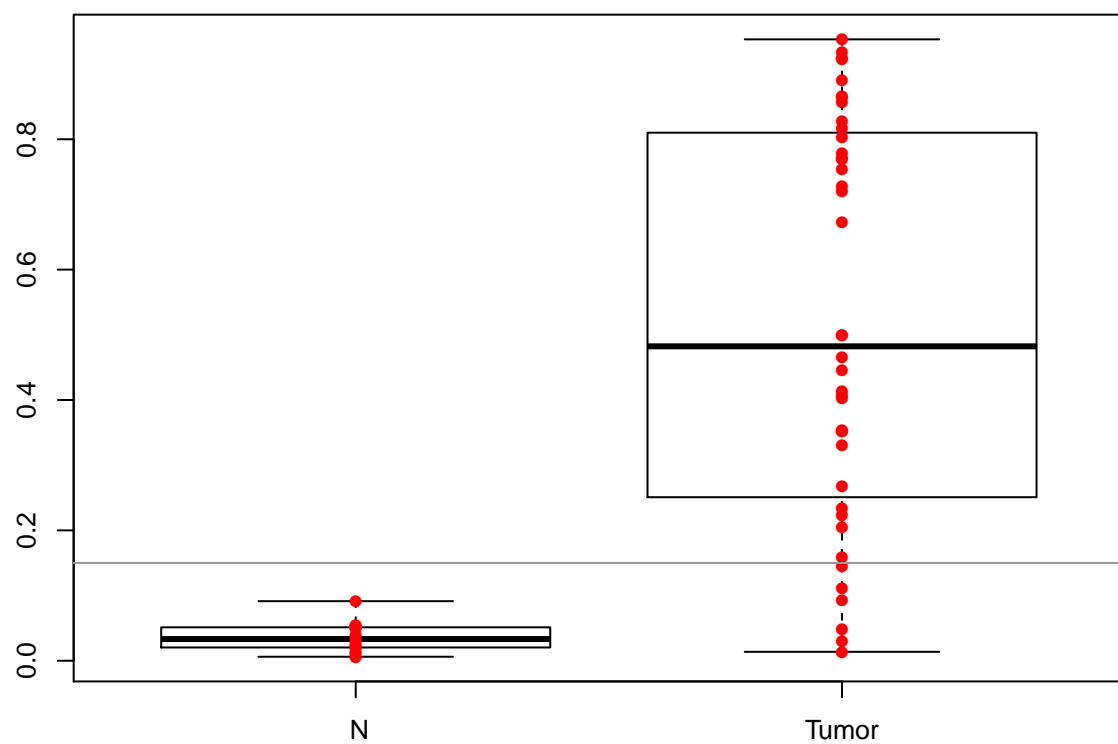T-test:  $1.127\text{e-}07$ **BNC1**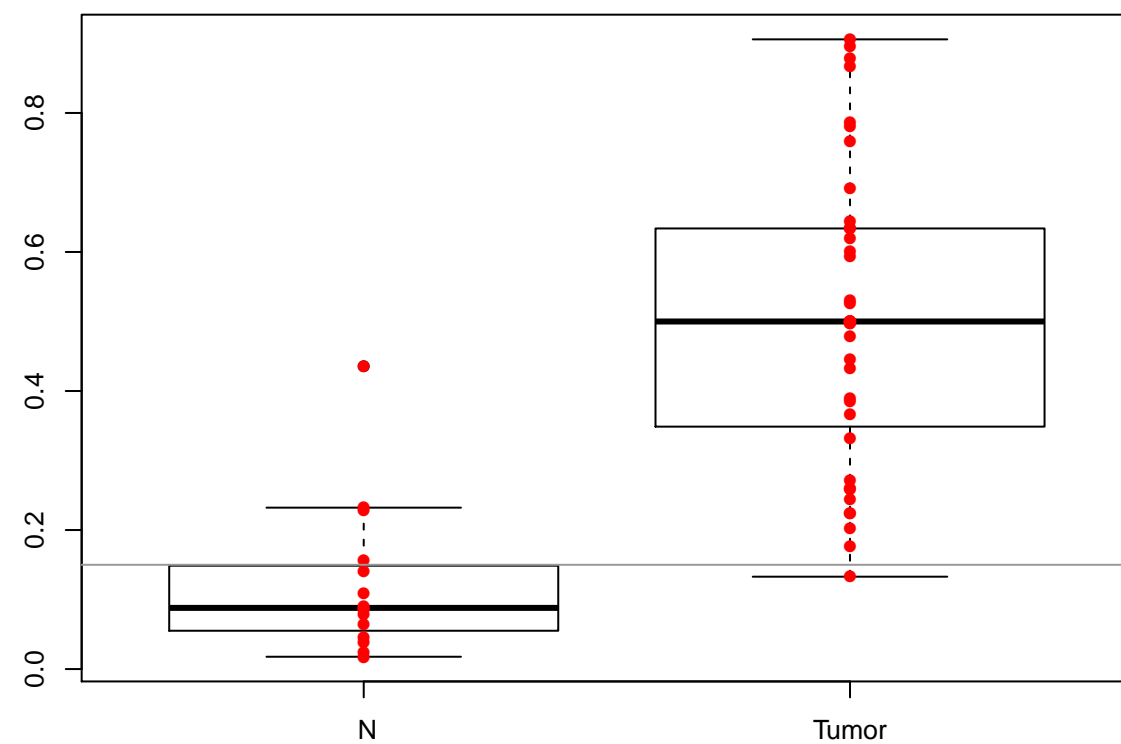T-test:  $2.562\text{e-}08$ **MAL**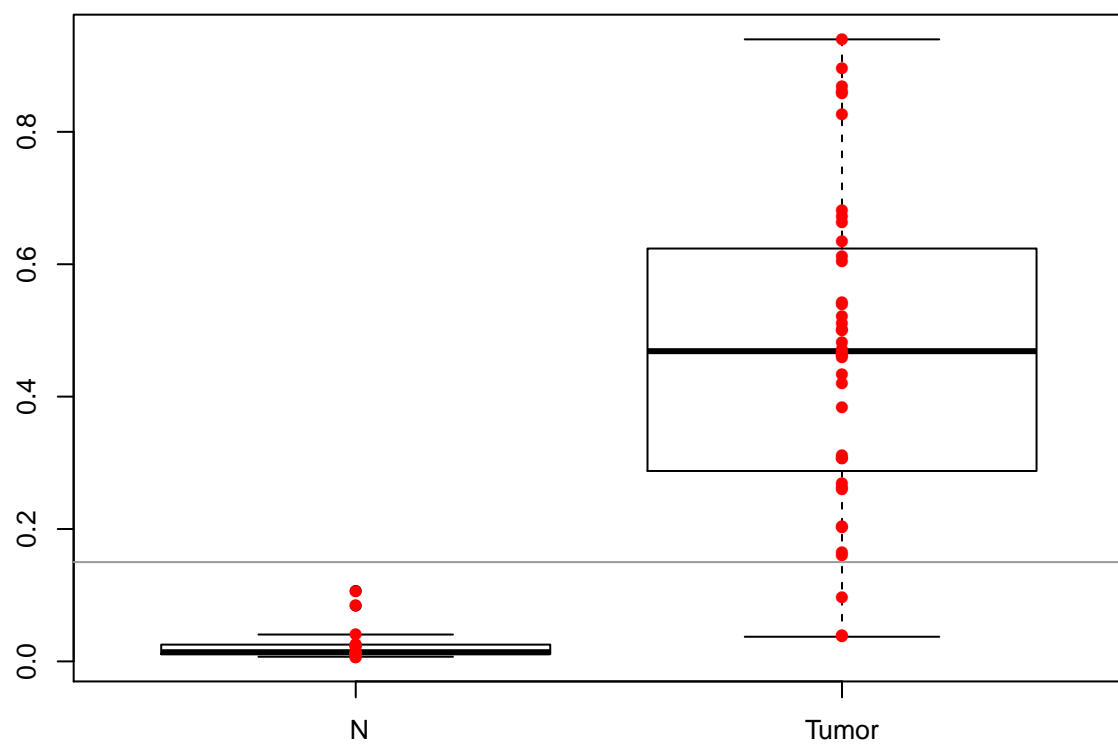T-test:  $3.072\text{e-}09$ **SFRP1**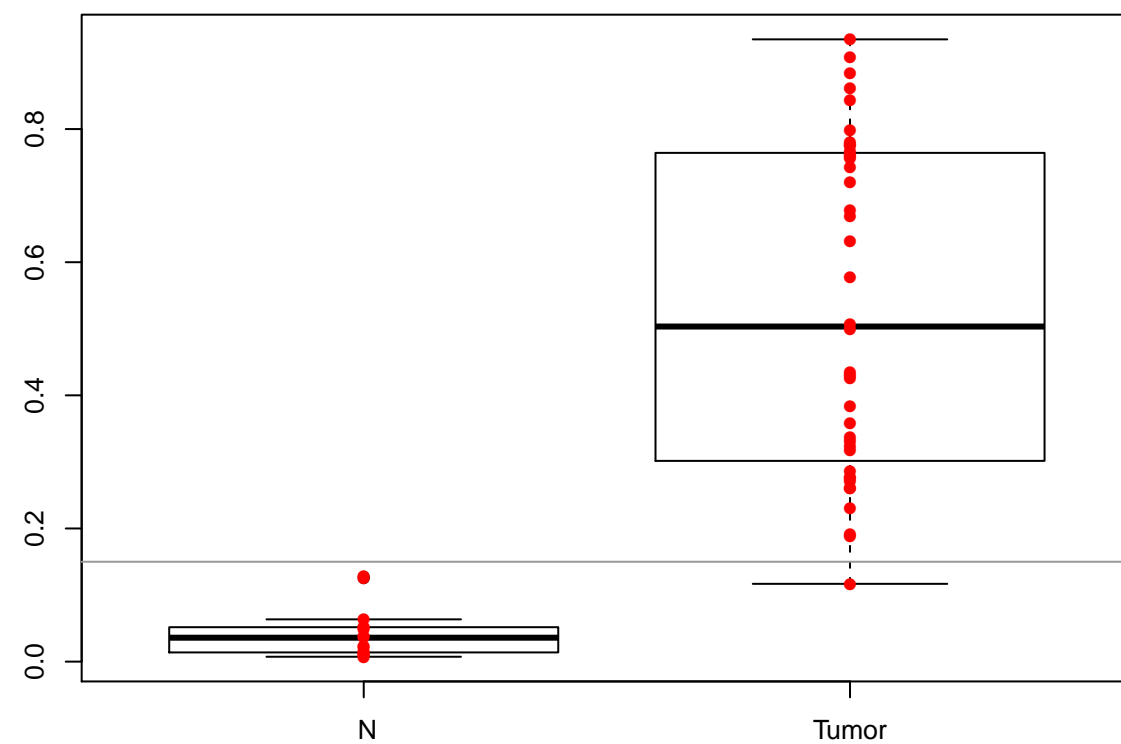T-test:  $4.84\text{e-}10$

Supplement: S1 Fig — Threshold for hypermethylation was set at 15% after comparing normal and cancerous samples in several genes. (PDF) [file pone.0133836.s001.pdf]

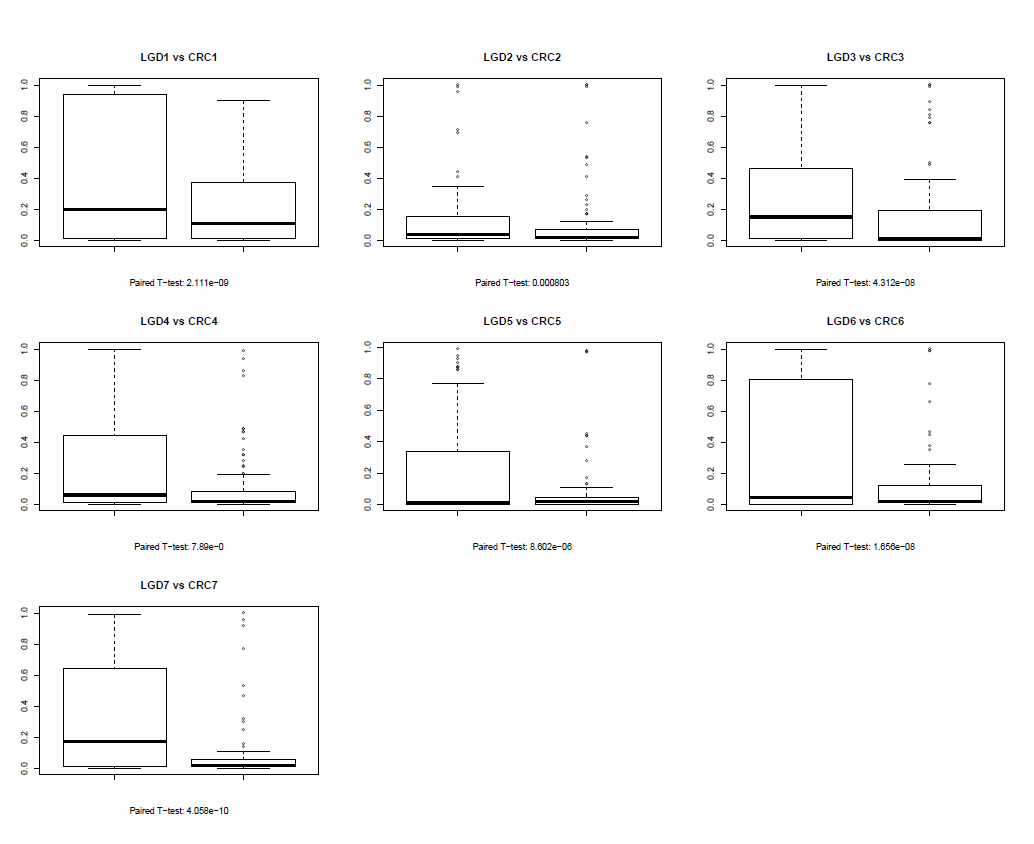

Supplement: S2 Fig — The mean DNA methylation percentage of methylated genes was significantly higher in LGD than in CRC. LGD: low-grade dysplasia, CRC: colorectal cancer (TIF) [file pone.0133836.s002.tif]

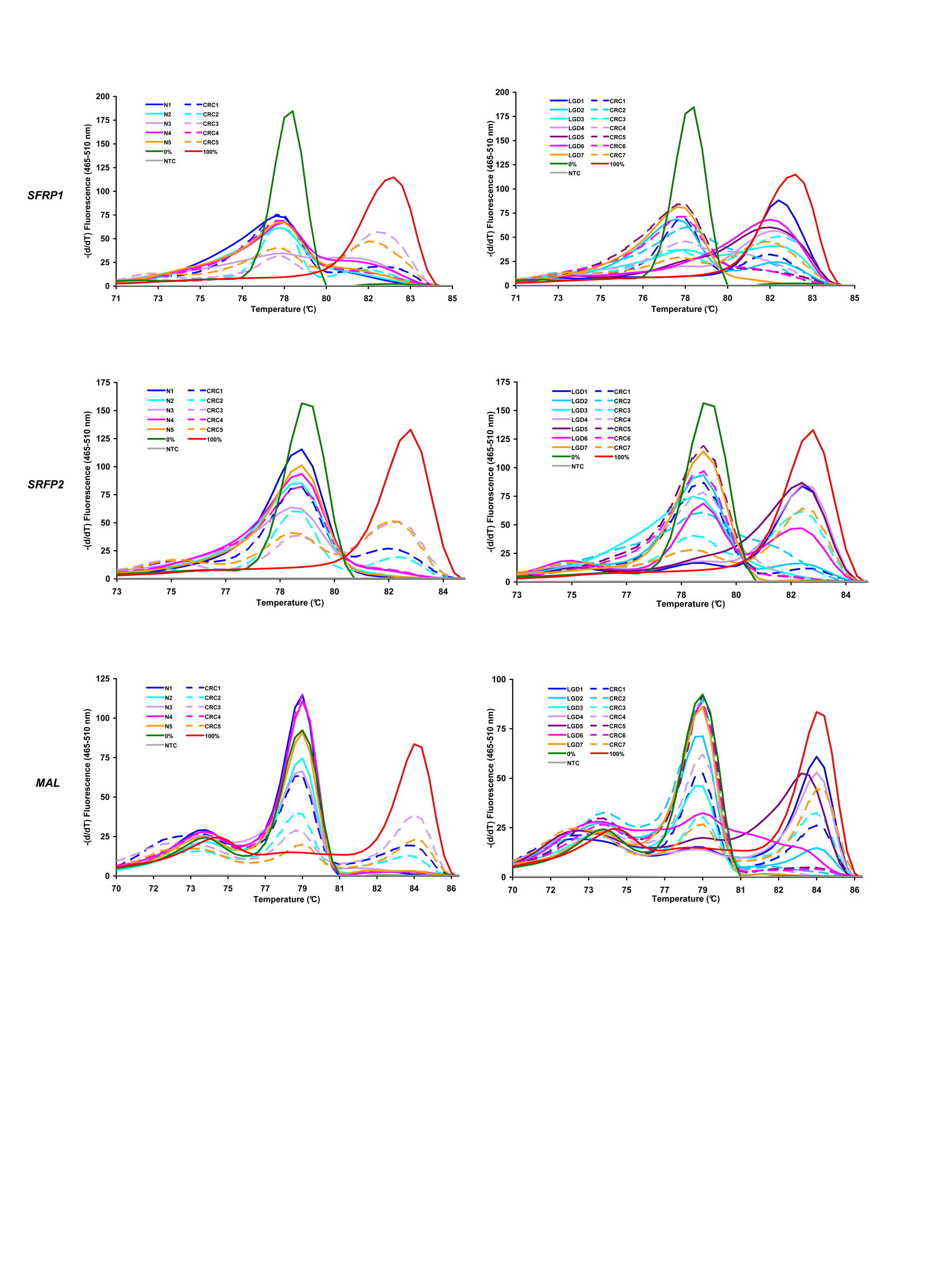

Supplement: S3 Fig — The inflection point on each standard melting curve is visualized here as a melting peak. Panels on the left show the degree of DNA methylation in normal (N) versus colorectal carcinoma (CRC) and panels on the right show that in low-grade dysplasia (LGD) versus CRC tissues. Sample pairs were obtained from the same patient. The melting peaks of methylated DNA standards (0% and 100%) and no template control (NTC) are also indicated for each gene studied. (TIF) [file pone.0133836.s003.tif]

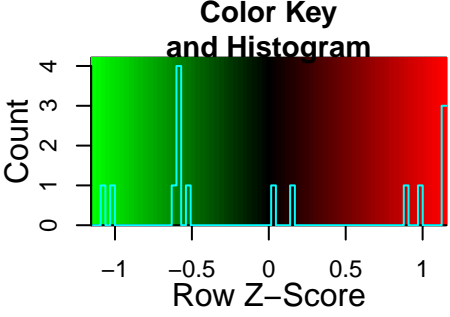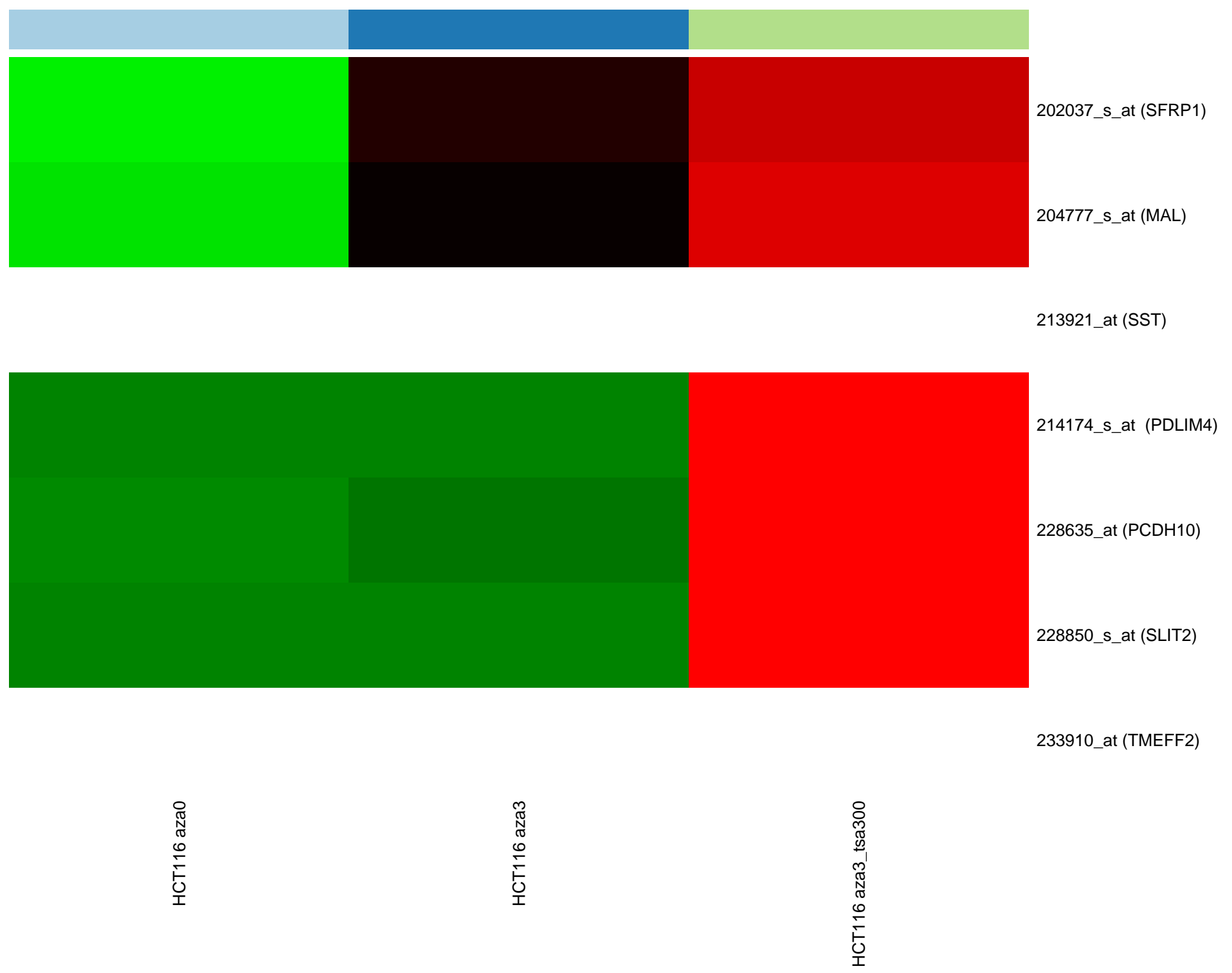

Supplement: S4 Fig — mRNA expression of some genes can be partly reversed by 5-aza-2’-deoxycytidine and trichostatin-A treatment on HCT116 cell line. HCT116 aza0: untreated, HCT116 aza3: 3 μM 5-AZA treatment for 72 hours, HCT116 aza3_tsa300: additional trichostatin-A (HDAC inhibitor) treatment. White squares indicate that gene expression was unchanged in that experiment. (PDF) [file pone.0133836.s004.pdf]
