# Supplementary material for: Comprehensive DNA Methylation Analysis Reveals a Common Ten-Gene Methylation Signature in Colorectal Adenomas and Carcinomas
Source: PLoS One. 2015 Aug 20;10(8):e0133836. doi: 10.1371/journal.pone.0133836 (PMC4546193; doi:10.1371/journal.pone.0133836)
Supplement: S1 Table — (DOCX) [file pone.0133836.s005.docx]

| **Gene Symbol** | **Gene name** |
| --- | --- |
| ADAMTS1 | ADAM metallopeptidase with thrombospondin type 1 motif, 1 |
| ALDH1A3 | Aldehyde dehydrogenase 1 family, member A3 |
| ALX4 | ALX homeobox 4 |
| APBA1 | Amyloid beta (A4) precursor protein-binding, family A, member 1 |
| APBA2 | Amyloid beta (A4) precursor protein-binding, family A, member 2 |
| APC | Adenomatous polyposis coli |
| ATM | Ataxia telangiectasia mutated |
| B4GALNT2 | Beta-1,4-N-acetyl-galactosaminyl transferase 2 |
| BAGE | B melanoma antigen |
| BMP3 | Bone morphogenetic protein 3 |
| BNC1 | Basonuclin 1 |
| CALCA | Calcitonin-related polypeptide alpha |
| CAV1 | Caveolin 1, caveolae protein, 22kDa |
| CCNA1 | Cyclin A1 |
| CD44 | CD44 molecule (Indian blood group) |
| CDH1 | Cadherin 1, type 1, E-cadherin (epithelial) |
| CDH13 | Cadherin 13, H-cadherin (heart) |
| CDKN2A | Cyclin-dependent kinase inhibitor 2A (melanoma, p16, inhibits CDK4) |
| CDKN2A | Cyclin-dependent kinase inhibitor 2A (melanoma, p16, inhibits CDK4) |
| CDX1 | Caudal type homeobox 1 |
| CDX2 | Caudal type homeobox 2 |
| CHFR | Checkpoint with forkhead and ring finger domains |
| CNR1 | Cannabinoid receptor 1 (brain) |
| CRABP1 | Cellular retinoic acid binding protein 1 |
| CTDSPL | CTD (carboxy-terminal domain, RNA polymerase II, polypeptide A) small phosphatase-like |
| CXCL12 | Chemokine (C-X-C motif) ligand 12 (stromal cell-derived factor 1) |
| DAB2IP | DAB2 interacting protein |
| DACT2 | Dapper, antagonist of beta-catenin, homolog 2 (Xenopus laevis) |
| DKK1 | Dickkopf homolog 1 (Xenopus laevis) |
| DKK2 | Dickkopf homolog 2 (Xenopus laevis) |
| DKK3 | Dickkopf homolog 3 (Xenopus laevis) |
| EPHB2 | EPH receptor B2 |
| EPHB4 | EPH receptor B4 |
| EXTL3 | Exostoses (multiple)-like 3 |
| EYA2 | Eyes absent homolog 2 (Drosophila) |
| FAM84A | Family with sequence similarity 84, member A |
| GALR2 | Galanin receptor 2 |
| H19 | H19, imprinted maternally expressed transcript (non-protein coding) |
| HIC1 | Hypermethylated in cancer 1 |
| HLTF | Helicase-like transcription factor |
| HNF1B | HNF1 homeobox B |
| HS3ST2 | Heparan sulfate (glucosamine) 3-O-sulfotransferase 2 |
| hsa-mir-342 | microRNA 342 |
| ID4 | Inhibitor of DNA binding 4, dominant negative helix-loop-helix protein |
| IGF2 | Insulin-like growth factor 2 (somatomedin A) |
| IGFBP3 | Insulin-like growth factor binding protein 3 |
| IGFBP7 | Insulin-like growth factor binding protein 7 |
| LRRC3B | Leucine rich repeat containing 3B |
| MAGEA1 | Melanoma antigen family A, 1 |
| MAL | Myelin and lymphocyte protein, T-cell differentiation protein |
| MCC | Mutated in colorectal cancers |
| MGMT | O-6-methylguanine-DNA methyltransferase |
| MLH1 | MutL homolog 1 (E. coli) |
| MSX1 | Homeobox, msh-like 1 |
| NEUROG1 | Neurogenin 1 |
| NID1 | Nidogen 1 |
| NKX2-5 | NK2 transcription factor related, locus 5 (Drosophila) |
| OPCML | Opioid binding protein/cell adhesion molecule-like |
| PAX2 | Paired box gene 2 |
| PCDH10 | Protocadherin 10 |
| PDLIM4 (RIL) | PDZ and LIM domain 4 |
| PGF | Placental growth factor |
| PRDM5 | PR domain containing 5 |
| PROM1 | Prominin 1 |
| PTGIS | Prostaglandin I2 (prostacyclin) synthase |
| PTGS2 | Prostaglandin-endoperoxide synthase 2 (prostaglandin G/H synthase and cyclooxygenase) |
| RAB32 | RAB32, member RAS oncogene family |
| RASSF1 | Ras association (RalGDS/AF-6) domain family member 1 |
| RBP1 | Retinol binding protein 1, cellular |
| RECK | Reversion-inducing-cysteine-rich protein with kazal motifs |
| RPRM | Reprimo, TP53 dependent G2 arrest mediator candidate |
| RUNX3 | Runt-related transcription factor 3 |
| SCNN1B | Sodium channel, nonvoltage-gated 1 beta |
| SFRP1 | Secreted frizzled-related protein 1 |
| SFRP2 | Secreted frizzled-related protein 2 |
| SFRP4 | Secreted frizzled-related protein 4 |
| SFRP5 | Secreted frizzled-related sequence protein 5 |
| SLC16A12 | Solute carrier family 16 (monocarboxylic acid transporters), member 12 |
| SLC5A8 | Solute carrier family 5 (iodide transporter), member 8 |
| SLIT2 | Slit homolog 2 (Drosophila) |
| SLIT3 | Slit homolog 3 (Drosophila) |
| SOCS1 | Suppressor of cytokine signaling 1 |
| SPARC | Secreted protein, acidic, cysteine-rich (osteonectin) |
| SST | Somatostatin |
| STK11 | Serine/threonine kinase 11 |
| TAC1 | Tachykinin 1 |
| TFAP2C | Transcription factor AP-2, gamma |
| TMEFF2 | Transmembrane protein with EGF-like and two follistatin-like domains 2 |
| UCHL1 | Ubiquitin carboxy-terminal hydrolase L1 |
| UGT1A1 | UDP glucuronosyltransferase 1 family, polypeptide A1 |
| VIM | Vimentin |
| WIF1 | Wnt inhibitory factor 1 |
| WNT5A | Wingless-related MMTV integration site 5A |
| WRN | Werner syndrome homolog (human) |
| WT1 | Wilms tumor 1 homolog |
| ZNF442 | Zinc finger protein 442 |
